# Model Evaluation in Generalized Structured Component Analysis Using Confirmatory Tetrad Analysis

**DOI:** 10.3389/fpsyg.2017.00916

**Published:** 2017-05-30

**Authors:** Ji Hoon Ryoo, Heungsun Hwang

**Affiliations:** ^1^Department of Educational Leadership, Foundations and Policy, University of VirginiaCharlottesville, VA, United States; ^2^Department of Psychology, McGill UniversityMontreal, QC, Canada

**Keywords:** confirmatory tetrad analysis, early childhood longitudinal study, generalized structured component analysis, model evaluation, structural equation modeling

## Abstract

Generalized structured component analysis (GSCA) is a component-based approach to structural equation modeling (SEM). GSCA regards weighted composites or components of indicators as proxies for latent variables and estimates model parameter via least squares without resorting to a distributional assumption such as multivariate normality of indicators. As with other SEM approaches, model evaluation is a crucial procedure in GSCA that is used to examine whether a hypothesized model is consistent with the data in hand. However, the few descriptive measures of model evaluation available for GSCA are limited to evaluating models in a more confirmatory manner. This study integrates confirmatory tetrad analysis (CTA) into GSCA for model evaluation or comparison. Although CTA has been used in factor-based SEM as an inferential statistic, CTA is actually more compatible with GSCA because it is completely free of the multivariate normality assumption. Utilizing empirical data collected for 18,174 students' social skills in an early childhood longitudinal study of 2010–11 kindergarten cohort, we demonstrate the capability and applicability of CTA in GSCA and compare its performance with existing measures for GSCA.

## Introduction

Generalized structured component analysis (GSCA; Hwang and Takane, 2004) is a component-based approach to structural equation modeling (SEM), where weighted composites or components of observed variables serve as proxies for latent variables. It estimates parameters via least squares (LS; Hwang and Takane, 2014) and thus does not require the multivariate normality assumption of indicators and seldom suffers from non-convergence, even in small samples. As will be shown shortly, GSCA expresses all sub-models into a single model formation, which in turn facilitates the derivation of a global optimization criterion that is consistently minimized to estimate parameters. Moreover, it can deal with more complex analyses (e.g., constrained multiple-group analysis, analysis of discrete indicators, etc.) in a straightforward and coherent manner, minimizing a single optimization criterion. Owing to its practical utility and flexibility, GSCA has already been applied to a wide range of psychological and bio-medical studies (e.g., Hwang et al., 2012, 2013; Jung et al., 2012; Romdhani et al., 2015).

Despite its growing popularity, GSCA currently relies on only a handful of descriptive measures for model evaluation and comparison, which includes FIT, AFIT, GFI, and SRMR (Hwang and Takane, 2014, Ch. 2). In this paper, we propose to apply confirmatory tetrad analysis (CTA; Bollen, 1990; Bollen and Ting, 1993, 1998, 2000; Hipp and Bollen, 2003) to GSCA as an additional and powerful model evaluation tool. In particular, we employ CTA to find the best fitting model among a pool of GSCA models as an inferential statistic. The current measures for GSCA are based on the difference between the fitted model and sample data (individual-level raw data or variances and covariances). Instead, CTA utilizes a number of so-called vanishing tetrads, which will be described shortly, to ensure that the test statistic in CTA is not based on the difference between the model and the data but the difference between vanishing tetrads from the model and the sample variance covariance matrix.

The paper is organized as follows: First, GSCA and the use of its fit indexes as descriptive measures of model evaluation are reviewed. Second, it introduces CTA as a model evaluation tool for GSCA, showing the relaxation of the normality condition in CTA. Last, it demonstrates the usefulness of CTA for model comparisons in GSCA using data on children's social skills extracted from an early childhood longitudinal study—Kindergarten: 2011 (ECLS-K: 2011). The final section discusses CTA's compatibility and applicability in GSCA.

## Methods

### Generalized structured component analysis (GSCA)

#### Model specification

**Figure 1:**
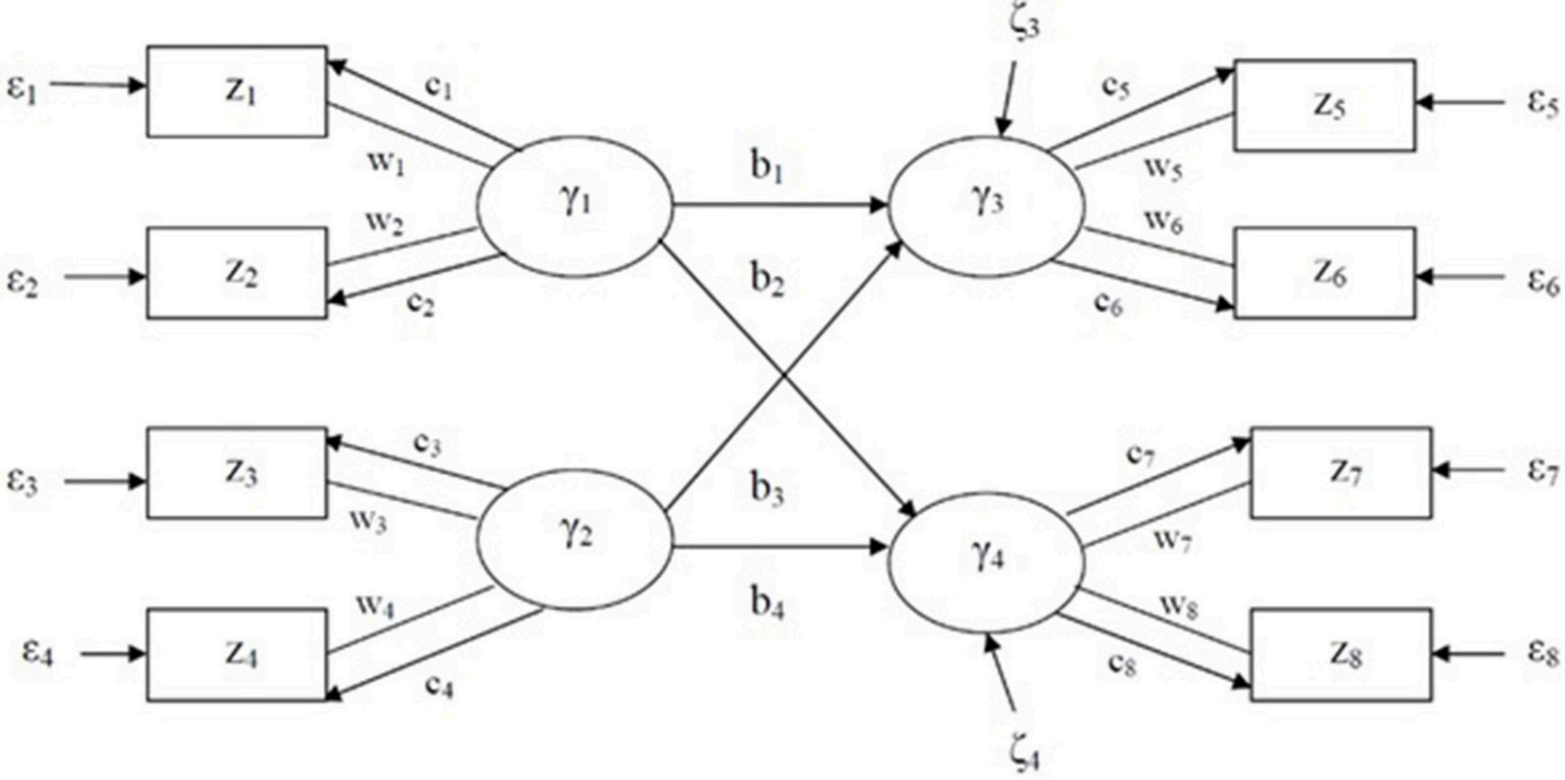
Generalized structured component analysis model consisting of measurement, structural, and weight models.

As stated earlier, GSCA is a component-based approach to SEM. It involves three sub-models: measurement, structural, and weighted relation models. The first two models are the same as those used in the LISREL model (Jöreskog, 1973, 1977, 1978), namely the measurement and structural models. The weighted relation model is used to define a latent variable as a weighted composite or component of indicators. These sub-models can be written in matrix form as follows:

$$\begin{array}{l} \text{Measurement model}:\;z=C^{T}\gamma \;+\;\varepsilon \\ \text{Structural model}:\;\gamma =B^{T}\gamma \;+\;\zeta \\ \text{Weighted relation model}:\;\gamma =W^{T}z \end{array}$$

where *z* is a *J* by 1 vector of indicators, γ is a *P* by 1 vector of latent variables, *C* is a *P* by *J* matrix of loadings, *B* is a *P* by *P* matrix of path coefficients, *W* is a *J* by *P* matrix of component weights, ε is a *J* by 1 vector of the residuals of indicators, and ζ is a *P* by 1 vector of the residuals of latent variables, where the superscript *T* is for a transpose matrix. As described in Figure 1, all notations except *w*_*i*_ for *i* = 1, …, 8 are the same as in the LISREL model. Latent variables, γ_*j*_ for *j* = 1, …, 4, are linear combinations of two indicators with weights, for example, γ_1_ = *w*_1_*z*_1_ + *w*_2_*z*_2_.

#### Estimation

GSCA estimates model parameters, including weights (*W*), path coefficients (*B*), and loadings (*C*), by minimizing the sum of the squares of the residuals, *e*_*i*_, i.e., consistently minimizing a single LS criterion defined by:

$$\begin{array}{l} \Phi \;=\;\overset{N}{\underset{i=1}{\sum }}e_{i}^{T}e_{i}\;=\;\overset{N}{\underset{i=1}{\sum }}{\left(V^{T}z_{i}-A^{T}W^{T}z_{i}\right)}^{T}\left(V^{T}z_{i}-A^{T}W^{T}z_{i}\right), \end{array}$$

where $A=\left[\begin{array}{c} C^{T}\\ B^{T} \end{array}\right]$ and *N* is the sample size. To make the scaling in indicators and latent variables consistent, it is assumed that both indicators and latent variables are standardized. In addition, standard errors of parameter estimates are computed using the bootstrap method (Efron, 1979, 1982). More details of the computation and algorithms involved are available in Hwang and Takane (2014, Ch. 2).

#### Model evaluation

As stated earlier, the model evaluation tools in GSCA include FIT (Henseler, 2012), Adjusted FIT (Hwang et al., 2007), GFI (Jöreskog and Sörbom, 1986), and SRMR (Hwang, 2008) measures for the overall model fit. FIT is defined as:

$$\begin{array}{l} FIT\;=\;1-\left[SS\left(ZV-ZWA\right)/SS\left(ZV\right)\right]\;=\;\frac{1}{T}\overset{T}{\underset{t=1}{\sum }}R_{t}^{2}, \end{array}$$

where $R_{t}^{2}$ is the *R*-squared value of each indicator or latent variable, and *T* is the total number of indicators and latent variables. The values of FIT range from 0 to 1 and can be interpreted as the variance accounted for by the model specification; the larger the value, the more the model's variance is explained as in linear regression.

Adjusted FIT (AFIT) is defined as:

$$\begin{array}{l} AFIT\;=\;1-\left(1-FIT\right)\frac{d_{0}}{d_{1}}, \end{array}$$

where *d*_0_ = *N* · *J* is the number of degrees of freedom for the null model (*W* = 0 and *A* = 0) and *d*_1_ = *N* · *J* − δ is the number of degrees of freedom for the model being compared, where δ is the number of free parameters. The model that maximizes AFIT can be regarded as the most appropriate among competing models.

Let *S* and $\hat{\Sigma }$ denote the sample covariance matrix and the model-implied covariance matrix evaluated at the LS estimates of parameters. Let *s*_*jq*_ and ${\hat{\sigma }}_{jq}$ denote the *jq* th elements in *S* and $\hat{\Sigma }$, respectively. Then the GFI and SRMR are calculated by:

$$\begin{array}{lll} GFI & = & 1-\frac{trace{\left(S-\hat{\Sigma }\right)}^{2}}{trace\left(S^{2}\right)}\\ SRMR & = & \sqrt{2\overset{J}{\underset{j=1}{\sum }}\overset{j}{\underset{q=1}{\sum }}\frac{{\left(\left(s_{jq}-{\hat{\sigma }}_{jq}\right)/\left(s_{jj}s_{qq}\right)\right)}^{2}}{J\left(J+1\right)}}. \end{array}$$

In general, GFI values close to 1 and an SRMR close to 0 is considered indicative of a good fit. In addition to the global fit indices, there are local fit indices such as FIT_M_, and FIT_S_ in GSCA. FIT_M_ (and FIT_S_) indicate how much the variance of indicators (and latent variables) is accounted for by a measurement (and a structural) model. These fits can also be interpreted in a similar way to that used in FIT. Here, the local fits were not considered but instead composite reliability was used (Werts et al., 1974) as a local fit when interpreting the factor reliability in the Results section. The composite reliability, ρ_*p*_, is defined by:

$$\begin{array}{l} \rho_{p}\;=\;\frac{{\left(\overset{J_{p}}{\underset{j=1}{\sum }}c_{pj}\right)}^{2}}{{\left(\overset{J_{p}}{\underset{j=1}{\sum }}c_{pj}\right)}^{2}+\overset{J_{p}}{\underset{j=1}{\sum }}\left(1-c_{pj}^{2}\right)} \end{array}$$

where *c*_*pj*_ is the loading value for an indicator, *z*_*pj*_, and *J*_*p*_ is the number of indicators for the *p* th latent variable.

### Confirmatory tetrads analysis (CTA)

A tetrad approach using the difference in the products of certain pairs of the covariances (or correlations) of observed variables was proposed by Glymour et al. (1987) as a method to search for a model consistent with the covariance matrix of observed variables. Their focus was on applying exploratory tetrad analysis (ETA) to search for a good match to the tetrads of the observed variables. A few years later, Bollen and Ting (1993) proposed the use of a CTA to test one or several specific models, utilizing vanishing tetrads (Bollen, 1990) that will be discussed shortly. CTA has been utilized within ML-based SEM (Bollen and Ting, 2000) and also applied in partial least squares (PLS; Wold, 1966, 1973, 1982; Gudergan et al., 2008). However, CTA in ML-based SEM requires a multivariate normality assumption to obtain model-implied variance-covariance matrix, whereas CTA in PLS does not take into account model specifications simultaneously. In GSCA, CTA can be used without the normality assumption, yielding a model evaluation tool that utilizes all of the model specifications.

As a model evaluation method, CTA is comparable to the likelihood ratio difference test (LRDT). While LRDT does not work for models that are non-identifiable models, not-convergent, or not-nested (parameter-wise), CTA is applicable to some of these models and, furthermore, can also be applied to evaluate each of the measurement and structural models in SEM. This flexibility holds even when CTA is applied to GSCA utilizing the LS estimation method.

**Figure 2:**
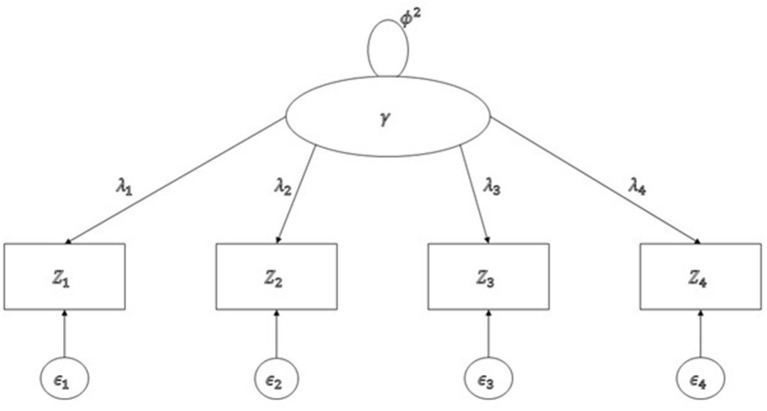
A single factor model with four observed variables.

A tetrad is defined as a form of four covariances of population covariance matrix (∑) as follows: τ_*ijkl*_ = σ_*ij*_σ_*kl*_ − σ_*ik*_σ_*jl*_. It is possible that τ_*ijkl*_ = 0, when it is called a *vanishing* tetrad. For example, if we consider four variables in a single factor model, as in Figure 2, there are only three vanishing tetrads, namely τ_1234_ = σ_12_σ_34_ − σ_13_σ_24_, τ_1342_ = σ_13_σ_42_ − σ_14_σ_32_, and τ_1423_ = σ_14_σ_23_ − σ_12_σ_43_, because all the product terms of covariances in the non-redundant tetrads are equal to $\lambda_{1}\lambda_{2}\lambda_{3}\lambda_{4}\varphi^{2}$ in Figure 2, where ϕ^2^ = var(γ).

Similar to the chi-square test in ML-based SEM, CTA is a hypothesis test using vanishing tetrads implied by a model, which means that we need a test statistic and its distribution to enable us to judge acceptance or rejection. First, CTA examines whether at least one vanishing tetrad exists based on a given model specification. The set of vanishing tetrads forms a null hypothesis such that if a model is correctly specified, the vanishing tetrads from the model-implied covariance should be zero when evaluated with the given data. This is an analog for minimizing the fit function generated by the difference between the sample variance-covariance and model-implied variance-covariance matrices. Thus, rejecting the null hypothesis means that the hypothesized model is misspecified. More specifically, in the hypothesis test, CTA identifies the vanishing tetrads in a model (i.e., *A* is an indicator matrix of the vanishing tetrad values for a given model specification), computes all of the vanishing tetrad values from the sample covariance matrix (i.e., $\hat{\tau }$ is the sample vector of vanishing tetrad values), and finds the asymptotic covariance matrix of the sample estimates of the tetrads (i.e., ${\hat{\Sigma }}_{\hat{\tau }}$ is the covariance matrix, that is, ${\hat{\Sigma }}_{\hat{\tau }}=A\cdot {\left(\frac{\partial \tau }{\partial \sigma }\right)}^{T}\cdot \Sigma_{S}\cdot \left(\frac{\partial \tau }{\partial \sigma }\right)\cdot A^{T}$, where Σ_*S*_ is the estimated asymptotic covariance matrix of the sample covariances). The test statistic in CTA is then defined as $T=n{\hat{\tau }}^{T}A^{T}{\hat{\Sigma }}_{\hat{\tau }}^{-1}A\hat{\tau }\sim \;\chi_{df}^{2}$, where *df* is the number of vanishing tetrads (Bollen, 1990) and its modified tetrad test statistic is defined as $T_{1}=n{\hat{\tau }}^{T}{\hat{\sum }}_{\hat{\tau }1}^{-1}\hat{\tau }$, where ${\hat{\sum }}_{\hat{\tau }1}=diag\left[{\left(\frac{\partial \tau }{\partial \sigma }\right)}^{T}\sum_{S}\left(\frac{\partial \tau }{\partial \sigma }\right)\right]$ (Johnson and Bodner, 2007).

When two models are compared using CTA, we begin by finding the vanishing tetrads on each of the given models by forming the implied covariance matrix and estimating all of the tetrads. Next, we identify a set of non-redundant vanishing tetrads to calculate ${\hat{\sum }}_{\hat{\tau }}$ (or ${\hat{\sum }}_{\hat{\tau }1}$) for each given model, yielding different results from the selection. Lastly, we compute the test statistics, *T* (or *T*_1_) for each given model, thus providing the result of the hypothesis test. In this model comparison, if the vanishing tetrads in Model 2 are a strict subset of the vanishing tetrads in Model 1, then Model 2 is said to be tetrad-nested in Model 1. As with LRDT, we then compute Δ*T* = *T*_1_ − *T*_2_ and compare it in the form $\chi_{df_{1}-df_{2}}^{2}$, which allows us either to accept or to reject the hypothesis. “Rejection” means that the less restrictive model (Model 2) has a better fit than the more restrictive model (Model 1). It should be noted that CTA works only for two tetrad-nested models.

### Procedure for model evaluation using CTA in GSCA

#### Step 1: model-implied correlation matrix in GSCA

When a hypothesized model and its sample data are available, we can fit GSCA to the sample data and obtain the results including model fit indexes and parameter estimates. In GSCA, as stated earlier, all indicators and latent variables are typically assumed to be standardized (Hwang and Takane, 2014), which allows us to obtain the model-implied correlation matrix for CTA. All analyses obtaining the model-implied correlation matrix were conducted using the R package known as gesca (Hwang et al., 2016).

#### Step 2: confirmatory tetrad analysis for a single model

Based on the model-implied correlation matrix from Step 1, we can now conduct a hypothesis testing to determine if the model fits well to the given data. Although the hypothesis test is clear enough, it is well known that the chi-square statistic is sensitive to a large sample size because the test statistic, $T=n{\hat{\tau }}^{T}A^{T}{\hat{\Sigma }}_{\hat{\tau }}^{-1}A\hat{\tau }$, will be inflated by *n* and thus trivial differences in the sample tetrad values from vanishing tetrads can lead to significant differences in the results (Bollen and Ting, 2000). Although there is no specific rule of thumb regarding what constitutes a large sample, “typical” sample sizes in SEM studies are 200–300 (Kline, 2016). Thus, a sample size larger than 1,000 would be considered large, rendering hypothesis testing not plausible. On the other hand, analogous to the likelihood ratio difference test (LRDT) with a chi-square distribution (Collins and Lanza, 2010; Kline, 2016), the chi-square distribution for $T=n{\hat{\tau }}^{T}A^{T}{\hat{\Sigma }}_{\hat{\tau }}^{-1}A\hat{\tau }$ is likely to be approximated reasonably well when Δ*T* = *T*_1_ − *T*_2_ has relatively few degrees of freedom, *df* = *df*_1_ − *df*_2_.

#### Step 3: model comparison using CTA

As noted in Bollen and Ting (1993) and Hipp and Bollen (2003), it is common to have redundant vanishing tetrads in many models. However, these redundant vanishing tetrads prevent us from correctly counting the number of degrees of freedom. For example, three vanishing tetrads shown in Figure 2 are redundant because σ_12_σ_34_ = σ_13_σ_24_ in τ_1234_ and σ_14_σ_32_ = σ_13_σ_42_ in τ_1342_ yield σ_14_σ_23_ = σ_12_σ_43_ in τ_1423_. To avoid any possibility that the result is contaminated due to these redundant vanishing tetrads, Hipp and Bollen (2003) recommended randomly selecting sets of vanishing tetrads multiple times. The tetrad command in Stata (StataCorp, 2013) uses the sweep operators originally designed (or used) to produce a generalized inverse (Goodnight, 1979) to identify sets of non-redundant vanishing tetrads (Hipp and Bollen, 2003; Hipp et al., 2005). When fitting CTA in Stata, researchers are allowed to specify a desired number of replications when randomizing the sets of non-redundant vanishing tetrads. By comparing Δ*T* = *T*_1_ − *T*_2_ in the distribution, $\chi_{df_{1}-df_{2}}^{2}$, we can decide which hypothesized model is well fitted from CTA.

### Empirical data

#### Early childhood longitudinal study

The Early Childhood Longitudinal Study, Kindergarten cohort: 2011 (ECLS-K: 2011) was run by the U.S. Department of Education and was designed to provide a longitudinal, descriptive dataset of children's early school experiences from kindergarten through middle school. The children in the study were a nationally representative sample of kindergarteners in 2010-2011, including children in both public and private school across the United States. Descriptive information gathered included aspects of development, home environment, and school environment, allowing researchers to examine how family, school, and individual factors are associated with subsequent school performance (http://nces.ed.gov/ecls/kindergarten.asp). The data used here is a “snapshot” of the ECLS-K data, gathered when the majority of the students in this cohort were in third grade. As public use data, the data used here are deidentified and decoded data and thus, no IRB required.

From the ECLS-K data, we extracted data on 18,174 children's social skill ratings (Gresham and Elliott, 1990), as measured by both teachers and parents. Teachers measured four scales [assigning scores ranging from 1 (low) to 4 (high)] for self-control (Mean = 3.216, *SD* = 0.623), interpersonal skills (Mean = 3.142, *SD* = 0.656), externalizing problems (Mean = 1.721, *SD* = 0.618), and internalizing problems (Mean = 1.539, *SD* = 0.505), while parents measured four scales (using the same range from 1 to 4) for self-control (Mean = 3.016, *SD* = 0.495), social interactions (Mean = 3.432, *SD* = 0.556), sad/lonely (Mean = 1.463, *SD* = 0.388), and impulsive/overactive behaviors (Mean = 1.869, *SD* = 0.663).

**Figure 3:**
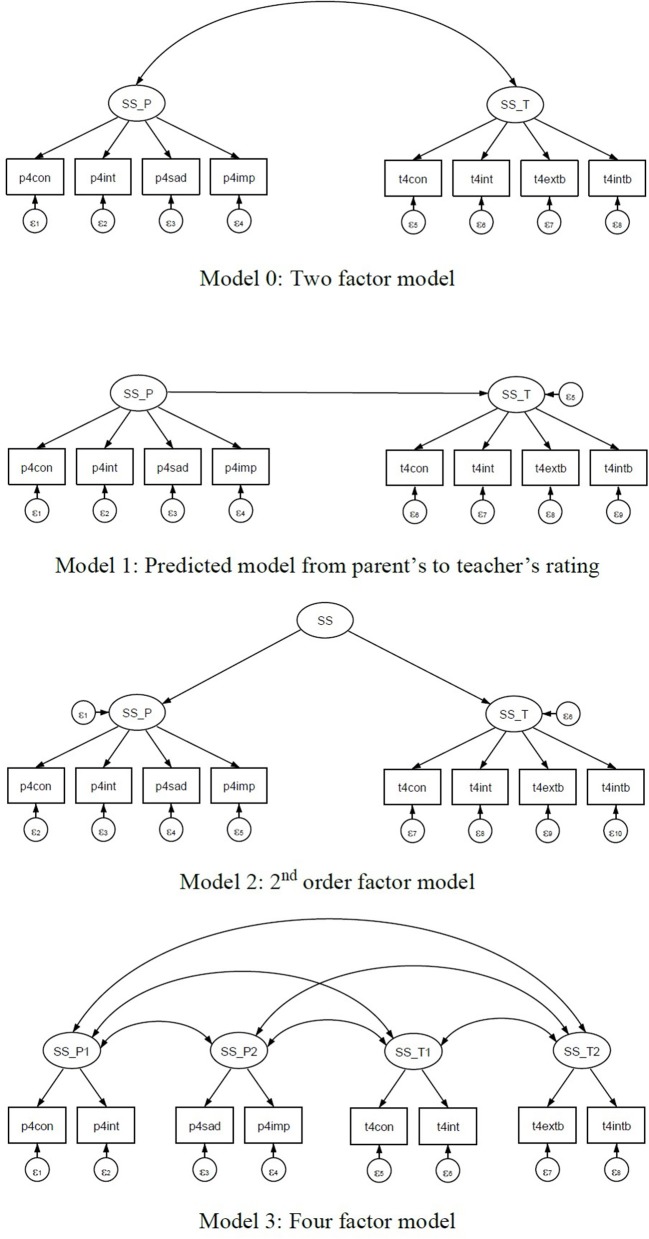
Four hypothetical models for children's social skills rated by both parent and teacher.

For this study examining the utility of the proposed new model evaluation tool introduced above, CTA in GSCA, we considered the following four models utilizing eight scales (see Figure 3), for instructional purposes. The four models considered would not be supported by any theory but should rather be considered hypothetically possible models. First, we considered a two-factor model (Model 0) based on the ratings of parents and teachers. Model 0 consists of the social skill factor of four items measured by teachers (self-control, interpersonal skills, externalizing problems, and internalizing problems), and the social skill factor of four items measured by parents (self-control, social interactions, sad/lonely, and impulsive/overactive). Both factors are correlated as shown in Figure 3. Second, we considered another two-factor model (Model 1) with directionality from parent-rated social skills to teacher-rated social skills. Both Model 0 and Model 1 are equivalent, with different structural models. Third, we considered a second-order factor model (Model 2) that imposed no constraints on the path coefficients from the second order social skill factor to the two first order social skills. Model 2 has more parameters than Model 0 and Model 1. Last, we considered a four-factor model (Model 3) that further divided two factors from the teacher-rated social skills (treating self-control and interpersonal skills as one factor and externalizing problems and internalizing problems as the other), and two factors from the parent-rated social skill (with one factor being self-control and social interactions and the other sad/lonely and impulsive/overactive).

To show the features of CTA, we chose two equivalent models (Model 0 and Model 1), and considered Model 2 as representing a non-tetrad-nested model compared with Model 3, and Model 3 as a tetrad-nested model within Models 0 and 1 (see Figure 3). In Step 2, we did not include the CTA test for a single model because our data, which included 18,174 first-grade students, meant that the sample size was too large for the chi-square test to be applied properly. In Step 3, we set up five replications, as used in Bauldry and Bollen (2016), to avoid any redundancy in vanishing tetrads.

## Results

In this section, we present three model comparisons using CTA in GSCA following the procedure described in the Method section. The three comparisons are based on the four hypothesized models for children's social skills depicted in Figure 3. The first example compares the two-factor model (Model 0) with the four-factor model (Model 3) of eight social skill items that are tetrad-nested. The second example compares the modified two-factor model, specifying a regression of teacher-rated social skills on parent-rated social skills (Model 1), with the four-factor model (Model 3). The third example applies CTA to the second-order factor model (Model 2) and the four-factor model (Model 3).

### Example 1: tetrad-nested model comparison I (Model 0 vs. Model 3)

In this example, the two-factor model (Model 0) is compared with the four-factor model (Model 3) for children's social skills as measured by teachers and parents. This type of comparison would be used in an exploratory factor analysis enumerating the number of factors. As a first step, we fit GSCA into both models using the gesca package in R to obtain the model-implied correlation matrices. As results of GSCA, we obtained the fit indexes, which for Model 0 were FIT (0.448), AFIT (0.448), GFI (0.989), and SRMR (0.106), while the fit indices for Model 3 were FIT (0.469), AFIT (0.469), GFI (0.975), and SRMR (0.238). Thus, Model 3 was better in terms of explaining the variance accounted for by the model specification (FIT and AFIT), whereas Model 0 was better in terms of minimizing the difference between the sample and model-implied variance and covariance (GFI and SRMR). However, the SRMRs of both models were outside the boundary of a good fit (<0.08; Hwang and Takane, 2014). We also examined the composite reliabilities (*r*; Werts et al., 1974), which were *r*_*P*_ = 0.77 and *r*_*T*_ = 0.88 in Model 0 and *r*_*P*1_ = 0.76, *r*_*P*2_ = 0.79, *r*_*T*1_ = 0.95, and *r*_*T*2_ = 0.79 in Model 3, where the subscripts P and T indicate the parent-rated and teacher-rated social skills, respectively. All of the *r*s are in the *good* range (>0.70; Nunnally, 1978). To obtain a more rational model comparison, we therefore moved on to CTA with the model-implied correlation matrices.

Before comparing Model 0 and Model 3, we ran CTA using the tetrad command in Stata as step 2. Although Model 0 (*T*_0_ = 1, 300, *p* < 0.05) and Model 3 (*T*_3_ = 662.4, *p* < 0.05) were not well fitted, this rejection of the null hypothesis is a common problem when sample sizes are large (here, *n* = 18, 174), as noted earlier. By conducting CTA for each model, we found that Model 0 includes 22 vanishing tetrads whereas Model 3 includes 15 vanishing tetrads, which shows that Model 3 is *possibly* tetrad-nested in Model 0 (see Table A.1 in Supplementary Material). The reason that we can only say that this is a possibility is that such a large number of vanishing tetrads would not guarantee the tetrad-nestedness when comparing both models simultaneously. In other words, it is possible that a vanishing tetrad in Model 0 would be a non-vanishing tetrad in Model 3. In step 3, we apply CTA with Model 0 and Model 3. Table A.1 indicates which tetrads are vanishing (1 in the fourth column) in each model but only lists the first 30 of the total 210 tetrads. As a default in the tetrad command, CTA examines the nestedness of two models and prints an error message if two models are not nested. Thus, if the output does not show any error message, then two models are nested. In this example, no error message was generated, thus confirming that Model 3 is nested in Model 0.

Next, these models are compared with five replications and the results summarized in Table 1. All five chi-square values were greater than 118.8 and significant at the level of 0.05. This signifies that Model 3 is better than Model 0. In other words, further classification of the two-factor model with the four-factor model yields a better understanding of children's social skills. This result is consistent with the FIT and AFIT values from GSCA obtained in step 1.

**Table 1:** Model comparison between Model 0 and Model 3, which are tetrad-nested.

**Rep**	**Model 0**			**Model 3**			**Model 3–Model 0**		
	**Chi-square**	***df***	***p*-value**	**Chi-square**	***df***	***p*-value**	**Chi-square**	***Df***	***p*-value**
1	964.0	21	0.000	845.2	16	0.000	118.8	5	0.000
2	1,000.0	21	0.000	538.8	14	0.000	489.1	7	0.000
3	1,000.0	21	0.000	603.8	14	0.000	407.5	7	0.000
4	959.0	22	0.000	651.9	15	0.000	307.1	7	0.000
5	1,200.0	20	0.000	830.4	15	0.000	385.0	5	0.000

### Example 2: tetrad-nested model comparison II (Model 1 vs. Model 3)

Model 1 specifies a regression of teacher-rated social skills on parent-rated social skills, which is equivalent to the model in Model 0, replacing a factor correlation. Although this is not going to be comparable in ML-based SEM, GSCA allows researchers to compare these models. The fit indices for Model 0 were FIT (0.448), AFIT (0.448), GFI (0.989), and SRMR (0.106), while the fit indices for Model 1 were FIT (0.465), AFIT (0.465), GFI (0.991), and SRMR (0.082). This shows that Model 1 was better than Model 0 for all four fit indices. The structural regression coefficient was 0.367 (*p* < 0.05 and *R*^2^ = 0.135), which means that 13.5% of the teacher-rated social skills were explained by parent-rated social skills. Thus, we can interpret the results to mean that teacher-rated social skills can be predicted by parent-rated social skills, although the effect was small (*R*^2^ = 0.135).

In this example, we compared Model 1 with the four-factor model (Model 3) for children's social skills measured by teachers and parents separately. In step 1, we fit GSCA into the data with both models compared. The fit indices for Model 1 were FIT (0.465), AFIT (0.465), GFI (0.991), and SRMR (0.082), while the fit indices for Model 3 were FIT (0.469), AFIT (0.469), GFI (0.975), and SRMR (0.238). Thus, Model 3 was slightly better for FIT and AFIT, whereas Model 1 was better for GFI and SRMR. However, the SRMRs of both models were still greater than the boundary for good fit (<0.08).

In step 2, we utilized the model-implied correlation obtained from GSCA to run CTA for the two models separately. Although neither Model 1 (*T*_1_ = 947.2, *p* < 0.05) nor Model 3 (*T*_3_ = 662.4, *p* < 0.05) were well fitted, as noted in Example 1 this rejection of the null hypothesis is common when sample sizes are large. We also obtained the results of CTAs for each model. Model 1 include 20 vanishing tetrads, while Model 3 had only 15, indicating that Model 3 is *possibly* tetrad-nested in Model 1 (see Table A.2 in Supplementary Material). The first 30 tetrads in Table A.2, and the lack of an error message in the CTA run confirmed that Model 3 is tetrad-nested in Model 1.

In step 3, we again compared these models with five replications; the results are summarized in Table 2. Although there are some variations in both chi-squares and dfs, all five replications indicated that Model 3 is better than Model 1; Model 3 with four factors for children's social skills is better fitted than the two-factor model with a structural regression from parent-rated social skills to teacher-rated social skills. Thus, this result is also consistent with those obtained for FIT and AFIT.

**Table 2:** Model comparison between Model 1 and Model 3, which are tetrad-nested.

**Rep**	**Model 1**			**Model 3**			**Model 3–Model 1**		
	**Chi-square**	***df***	***p*-value**	**Chi-square**	***df***	***p*-value**	**Chi-square**	***df***	***p*-value**
1	947.2	20	0.000	845.2	16	0.000	102.0	4	0.000
2	1,000.0	21	0.000	814.2	14	0.000	222.1	7	0.000
3	1,000.0	21	0.000	688.3	14	0.000	318.7	7	0.000
4	694.6	19	0.000	658.2	15	0.000	36.4	4	0.000
5	991.3	19	0.000	732.1	16	0.000	259.2	3	0.000

### Example 3: CTA with non-tetrad-nested models (Model 2 vs. Model 0 and Model 3)

In this example, we demonstrate both the advantage of CTA over the chi-square test in ML-based SEM and its slight disadvantage over FIT, AFIT, GFI, and SRMR in GSCA. This suggests that CTA is a useful model evaluation tool but not a panacea. When considering Model 2, we did not constrain the factor loadings from the second-order factor to two first-order factors. This relaxation caused non-convergence in the ML-based SEM, which prevented us from comparing Model 2 with the other models. However, there was no specific reason preventing us from obtaining a model fit because the model was over-identified.

We therefore moved on to fit GSCA to the data with a second-order factor model (Model 2). The result of step 1 indicated that the fit indices for Model 2 were much higher in FIT (0.525) and AFIT (0.525) than Model 0, but the fit indices for Model 2 were worse in GFI (0.959) and SRMR (0.118) than Model 0. This indicates that Model 2 was better in FIT and AFIT but Model 0 was better in GFI and SRMR. Although we did not find any superiority of Model 2 over Model 0 in terms of all four fit indexes (FIT, AFIT, GFI, and SRMR) in GSCA simultaneously, this could be a potential model for children's social skills rated by teachers and parents in GSCA. Next, we applied CTA using the tetrad command in Stata to determine whether Model 2 can be supported from the social skills data. In step 2, CTA provided the result that $T_{2}=702.3\sim \;\chi_{19}^{2}$ and *p* < 0.05. This shows that Model 2 is not supported from the data, although once again this finding is not plausible due to the large sample size. This shows that CTA is more applicable than the chi-square test in ML-based SEM.

By conducting CTA using the tetrad command in Stata, we then obtained the results of the CTAs for each model. Model 2 includes 19 vanishing tetrads, whereas Model 3 includes only 15 vanishing tetrads, which shows that Model 3 is *possibly* tetrad-nested in Model 2. However, when we compared the two models in step 3, the results showed that the two models are not tetrad-nested; with two non-vanishing tetrads, *t*_4,785_ and *t*_4,786_, in Model 2 out of the 210 tetrads identified as vanishing tetrads in Model 3, for example (see Table A.3 in Supplementary Material). This suggests that the total numbers of vanishing tetrads observed in step 1 do not guarantee the tetrad-nestedness between two models. Because none of these models is tetrad-nested to the other, Model 2 and Model 3 cannot be compared with CTA. Similarly, Model 0 was not tetrad-nested in Model 2. Thus, they cannot be compared with CTA. This result indicates that CTA is not a panacea for model evaluation.

## Discussion

The results of the CTA in GSCA were consistent with the FIT and AFIT indices in GSCA in the first two examples. Although GFI values were higher in the more restrictive models (Model 0 and Model 1) than in Model 3, all of the GFIs were greater than 0.95, which is in the good range. None of the four models showed SRMR <0.08, and thus we cannot derive definitive results from this demonstration. Likewise, although CTA can also be used as a model evaluation in GSCA, we were unable to conclude that CTA is more applicable to GSCA than FIT, AFIT, GFI, and SRMR because CTA does not work for non-tetrad nested models. This is analogous to the relationship between LRDT and fit indices such as AIC and BIC.

As in previous applications of CTA in ML-based SEM (Bauldry and Bollen, 2016), CTA is not a totally distribution-free method because it requires a model-implied correlation (or covariation) under the multivariate normality assumption. On the other hand, when CTA is used in GSCA, the model-implied correlation can be fitted without the need to make a distributional assumption, and hence the whole procedure for CTA does not require any distributional assumption. Moreover, one important benefit of CTA is that some models that have not been specifically identified can still be assessed based on their implied vanishing tetrads (Bollen and Ting, 2000) and not-convergent models can also be assessed, as shown in this paper.

In spite of the applicability of CTA, FIT, AFIT, GFI, and SRMR in GSCA, many underdeveloped areas in model selection remain in GSCA, including the provision of cutoffs for indexes and the inclusion of testing methods designed to select a better model, such as the likelihood ratio test. Along with CTA in GSCA, we plan to investigate the use of the rule of thumb in our future research, as suggested by Hu and Bentler (1999). Another possibly fruitful research topic is the application of CTA to measurement and structural models separately. Although we did not seek to apply CTA for each measurement and structural part of the model in this paper, CTA is applicable to parts of the model in GSCA, similar to its use by Gudergan et al. (2008) for PLS. This would ensure CTA is compatible with FIT_M_ and FIT_S_ as well as FIT.

### Limitations

Although Hwang and Takane (2014) provided guidance on how FIT and AFIT should be interpreted, they did not specify appropriate cutoff values. Thus, it was hard to determine whether or not the four models used in this study were in the good fit range as a single model. GFI indicated that all four of the models fell in the good fit range (>0.95), but SRMR indicated that all four were in the unacceptable range (>0.08). As noted above, as a future study we plan to conduct a simulation study to recommend cutoffs for use in model evaluations in GSCA, as Hu and Bentler (1999) and Cheung and Rensvold (2002) did for factor-based SEM.

Another limitation of the current study is the lack of a unified software package. To apply CTA in GSCA, we first had to fit each model using R's gesca package and compute the model-implied correlation matrix and then move on to utilize the matrix in the tetrad command in Stata. As a future study, we plan to develop a unified package in R. This package can then be used for conducting simulation studies to investigate the performance of CTA in GSCA under various experimental conditions.

## Concluding remarks

Both CTA and GSCA have been underused in spite of their applicability for both small and large samples, as well as for some under-identified models. The results of this study show great promise for developing a new approach that applies CTA to GSCA to extend its capability and applicability to the point that applied researchers will be able to use it in their studies.

## Author contributions

JR participated in the design, collected data, performed the statistical analysis, and drafted the manuscript. HH participated in the design, and drafted the manuscript. All authors read and approved the final manuscript.

### Conflict of interest statement

The authors declare that the research was conducted in the absence of any commercial or financial relationships that could be construed as a potential conflict of interest.
