# Supplementary material for: Model Evaluation in Generalized Structured Component Analysis Using Confirmatory Tetrad Analysis
Source: Front Psychol. 2017 May 30;8:916. doi: 10.3389/fpsyg.2017.00916 (PMC5447725; doi:10.3389/fpsyg.2017.00916)
Supplement: Supplementary file 1 [file Table1.DOCX]

**Appendix A.**

Table A.1.

*First 30 tetrads for Models 0 and 3 (1 signifies a vanishing tetrad and 0 a non-vanishing tetrad).*

| Model 0 | | | | | Model 3 | | | | |
| --- | --- | --- | --- | --- | --- | --- | --- | --- | --- |
| tetrad | residual | Avar | t-value | vanish | tetrad | residual | Avar | t-value | vanish |
| 1234 | 0.000 | 0.000 | -0.001 | 1 | 1234 | 0.120 | 0.000 | 22.007 | 0 |
| 1342 | 0.000 | 0.000 | 0.000 | 1 | 1342 | 0.000 | 0.000 | 0.000 | 1 |
| 1423 | 0.000 | 0.000 | 0.000 | 1 | 1423 | -0.120 | 0.000 | -57.490 | 0 |
| 1235 | 0.000 | 0.000 | 0.000 | 1 | 1235 | 0.015 | 0.000 | 3.756 | 0 |
| 1352 | 0.000 | 0.000 | 0.000 | 1 | 1352 | 0.000 | 0.000 | 0.000 | 1 |
| 1523 | 0.000 | 0.000 | 0.000 | 1 | 1523 | -0.015 | 0.000 | -7.133 | 0 |
| 1236 | 0.000 | 0.000 | 0.000 | 1 | 1236 | 0.015 | 0.000 | 3.756 | 0 |
| 1362 | 0.000 | 0.000 | 0.000 | 1 | 1362 | 0.000 | 0.000 | 0.000 | 1 |
| 1623 | 0.000 | 0.000 | 0.000 | 1 | 1623 | -0.015 | 0.000 | -7.133 | 0 |
| 1237 | 0.000 | 0.000 | 0.000 | 1 | 1237 | 0.020 | 0.000 | 5.026 | 0 |
| 1372 | 0.000 | 0.000 | 0.000 | 1 | 1372 | 0.000 | 0.000 | 0.000 | 1 |
| 1723 | 0.000 | 0.000 | 0.000 | 1 | 1723 | -0.020 | 0.000 | -9.988 | 0 |
| 1238 | 0.000 | 0.000 | 0.000 | 1 | 1238 | 0.020 | 0.000 | 5.016 | 0 |
| 1382 | 0.000 | 0.000 | 0.000 | 1 | 1382 | 0.000 | 0.000 | 0.000 | 1 |
| 1823 | 0.000 | 0.000 | 0.000 | 1 | 1823 | -0.020 | 0.000 | -9.968 | 0 |
| 1245 | 0.000 | 0.000 | 0.000 | 1 | 1245 | 0.015 | 0.000 | 3.759 | 0 |
| 1452 | 0.000 | 0.000 | 0.000 | 1 | 1452 | 0.000 | 0.000 | 0.000 | 1 |
| 1524 | 0.000 | 0.000 | 0.000 | 1 | 1524 | -0.015 | 0.000 | -7.134 | 0 |
| 1246 | 0.000 | 0.000 | 0.000 | 1 | 1246 | 0.015 | 0.000 | 3.759 | 0 |
| 1462 | 0.000 | 0.000 | 0.000 | 1 | 1462 | 0.000 | 0.000 | 0.000 | 1 |
| 1624 | 0.000 | 0.000 | 0.000 | 1 | 1624 | -0.015 | 0.000 | -7.134 | 0 |
| 1247 | 0.000 | 0.000 | 0.000 | 1 | 1247 | 0.020 | 0.000 | 5.029 | 0 |
| 1472 | 0.000 | 0.000 | 0.000 | 1 | 1472 | 0.000 | 0.000 | 0.000 | 1 |
| 1724 | 0.000 | 0.000 | 0.000 | 1 | 1724 | -0.020 | 0.000 | -9.989 | 0 |
| 1248 | 0.000 | 0.000 | 0.000 | 1 | 1248 | 0.020 | 0.000 | 5.019 | 0 |
| 1482 | 0.000 | 0.000 | 0.000 | 1 | 1482 | 0.000 | 0.000 | 0.000 | 1 |
| 1824 | 0.000 | 0.000 | 0.000 | 1 | 1824 | -0.020 | 0.000 | -9.969 | 0 |
| 1256 | 0.113 | 0.000 | 21.141 | 0 | 1256 | 0.172 | 0.000 | 27.031 | 0 |
| 1562 | 0.000 | 0.000 | 0.000 | 1 | 1562 | 0.000 | 0.000 | 0.000 | 1 |
| 1625 | -0.113 | 0.000 | -100.000 | 0 | 1625 | -0.172 | 0.000 | -180.000 | 0 |

*Note*: Every pair of *vanish* columns is one of (1,1), (1,0), and (0,0) in the first 30 tetrads out of 210 total tetrads: (1,1) indicates that the tetrad is vanishing on both models, (1,0) indicates that the tetrad is vanishing at Model 0 but not at Model 3, and (0,0) indicates that the tetrad is not vanishing on both models.
