# Supplementary material for: Model Evaluation in Generalized Structured Component Analysis Using Confirmatory Tetrad Analysis
Source: Front Psychol. 2017 May 30;8:916. doi: 10.3389/fpsyg.2017.00916 (PMC5447725; doi:10.3389/fpsyg.2017.00916)
Supplement: Supplementary file 3 [file Table3.DOCX]

**Appendix A.**

Table A.3.

*Tetrads for Models 2 and 3 (1 signifies a vanishing tetrad and 0 a non-vanishing tetrad).*

| Model 2 | | | | | Model 3 | | | | |
| --- | --- | --- | --- | --- | --- | --- | --- | --- | --- |
| tetrad | residual | Avar | t-value | vanish | tetrad | residual | Avar | t-value | vanish |
| 1234 | 0.000 | 0.000 | 0.000 | 1 | 1234 | 0.120 | 0.000 | 22.007 | 0 |
| 1342 | 0.000 | 0.000 | 0.000 | 1 | 1342 | 0.000 | 0.000 | 0.000 | 1 |
| 1423 | 0.000 | 0.000 | 0.000 | 1 | 1423 | -0.120 | 0.000 | -57.490 | 0 |
| 1235 | 0.000 | 0.000 | 0.000 | 1 | 1235 | 0.015 | 0.000 | 3.756 | 0 |
| 1352 | 0.000 | 0.000 | 0.000 | 1 | 1352 | 0.000 | 0.000 | 0.000 | 1 |
| 1523 | 0.000 | 0.000 | 0.000 | 1 | 1523 | -0.015 | 0.000 | -7.133 | 0 |
| 1236 | 0.000 | 0.000 | 0.000 | 1 | 1236 | 0.015 | 0.000 | 3.756 | 0 |
| 1362 | 0.000 | 0.000 | 0.000 | 1 | 1362 | 0.000 | 0.000 | 0.000 | 1 |
| 1623 | 0.000 | 0.000 | 0.000 | 1 | 1623 | -0.015 | 0.000 | -7.133 | 0 |
| 1237 | 0.000 | 0.000 | 0.000 | 1 | 1237 | 0.020 | 0.000 | 5.026 | 0 |
| 1372 | 0.000 | 0.000 | 0.000 | 1 | 1372 | 0.000 | 0.000 | 0.000 | 1 |
| 1723 | 0.000 | 0.000 | 0.000 | 1 | 1723 | -0.020 | 0.000 | -9.988 | 0 |
| 1238 | 0.000 | 0.000 | 0.000 | 1 | 1238 | 0.020 | 0.000 | 5.016 | 0 |
| 1382 | 0.000 | 0.000 | 0.000 | 1 | 1382 | 0.000 | 0.000 | 0.000 | 1 |
| 1823 | 0.000 | 0.000 | 0.000 | 1 | 1823 | -0.020 | 0.000 | -9.968 | 0 |
| 1245 | 0.000 | 0.000 | 0.000 | 1 | 1245 | 0.015 | 0.000 | 3.759 | 0 |
| 1452 | 0.000 | 0.000 | 0.000 | 1 | 1452 | 0.000 | 0.000 | 0.000 | 1 |
| 1524 | 0.000 | 0.000 | 0.000 | 1 | 1524 | -0.015 | 0.000 | -7.134 | 0 |
| 1246 | 0.000 | 0.000 | 0.000 | 1 | 1246 | 0.015 | 0.000 | 3.759 | 0 |
| 1462 | 0.000 | 0.000 | 0.000 | 1 | 1462 | 0.000 | 0.000 | 0.000 | 1 |
| ⁞ | ⁞ | ⁞ | ⁞ | ⁞ | ⁞ | ⁞ | ⁞ | ⁞ | ⁞ |
| 4578 | 0.000 | 0.000 | 0.000 | 1 | 4578 | 0.008 | 0.000 | 2.017 | 0 |
| **4785** | **0.000** | **0.000** | **-0.001** | **0** | **4785** | **0.000** | **0.000** | **0.000** | **1** |
| 4857 | 0.000 | 0.000 | 0.000 | 1 | 4857 | -0.008 | 0.000 | -2.694 | 0 |
| 4678 | 0.000 | 0.000 | 0.000 | 1 | 4678 | 0.008 | 0.000 | 2.017 | 0 |
| **4786** | **0.000** | **0.000** | **-0.001** | **0** | **4786** | **0.000** | **0.000** | **0.000** | **1** |
| 4867 | 0.000 | 0.000 | 0.000 | 1 | 4867 | -0.008 | 0.000 | -2.694 | 0 |
| 5678 | 0.000 | 0.000 | 0.000 | 1 | 5678 | 0.109 | 0.000 | 19.239 | 0 |
| 5786 | 0.000 | 0.000 | 0.000 | 1 | 5786 | 0.000 | 0.000 | 0.000 | 1 |
| 5867 | 0.000 | 0.000 | 0.000 | 1 | 5867 | -0.109 | 0.000 | -35.057 | 0 |

*Note*: **Bold** indicates tetrads breaking the tetrad-nestedness between Model 2 and Model 3 as an example.
